# Preoperative evaluation of bone quality for dental implantation using an ultrasound axial transmission device in an ex vivo model

**DOI:** 10.1002/cre2.59

**Published:** 2017-06-09

**Authors:** Shinsuke Okada, Akira Kawano, Hiroshi Oue, Yosuke Takeda, Miyuki Yokoi, Katsunori Koretake, Kazuhiro Tsuga

**Affiliations:** ^1^ Department of Advanced Prosthodontics, Graduate School of Biomedical & Health Sciences Hiroshima University Hiroshima Japan

**Keywords:** axial transmission device, bone quality, dental implantation, ultrasound

## Abstract

This study investigated the clinical utility of an ultrasound axial transmission device in preoperative evaluation of bone quality for dental implantation, by clarifying the relationship between cortical bone speed of sound (cSOS), insertion torque values (ITV), and implant stability quotient (ISQ) in porcine femur bone. Eleven fresh porcine femurs, without soft tissue, were prepared. The cSOS of these bones were measured using the axial transmission device. Bone mineral density (BMD) and porosity (Po) were measured in cortical bone samples obtained from the region of ultrasound measurements by X‐ray microcomputed tomography. Thirty‐three implants were inserted into these samples (three implants per bone sample), and ITV and ISQ were measured for all implants. Then, cortical bone thickness (CbTh) of the area for implantation was measured for all implants using a micrometer. The mean cSOS was 3962 m/s; mean BMD and Po were 0.822 g/cm2 and 0.185%, respectively. cSOS and BMD values were positively correlated, and cSOS values and Po values were negatively correlated. Mean ITV, ISQ, and CbTh were 37.95 Ncm, 71.172, and 2.869 mm, respectively. There was a positive correlation between cSOS values and ISQ values. The cSOS of each bone did not correlate with ITV for all of the bone samples. However, when the CbTh ranges from 3.0 to 3.5 mm, ITV are correlated with cSOS. These findings suggest that cSOS, which reflects the cortical bone quality, may be clinical utility as a preoperative diagnosis of the implant.

## 1 INTRODUCTION

Primary stability is regarded as an important factor for success in dental implant treatments and one of the most important prerequisites for osseointegration (Beer, Gahleitner, Holm, Tschabitscher, & Homolka, 2003; Gömez‐Polo et al., 2016). It has been shown that primary stability is influenced by the geometry of an implant (i.e., the length, diameter, shape, and thread), the placement technique, and bone quantity and quality in the area targeted for implantation (Beer et al., 2003; Klein, Grötz, Manefeld, Kann, & Al‐Nawas, 2008; Toyoshima et al., 2011). Many studies have shown satisfactory survival rates of dental implants placed into the mandible, with a tendency for lower survival rates of implants placed in the maxilla or into augmented bone with a poorer bone quality than that of mandibular bone (Lindhe, Obrant, & Petersson, 2004).

Quantitative computed tomography (qCT) has been used as a traditional preoperative evaluation for measuring bone quality. Bone mineral density (BMD) values measured by qCT have been shown to be capable of predicting primary stability (Beer et al., 2003; Turkyilmaz, Tumer, Ozbek, & Tözüm, 2007). However, qCT is not suitable for repeated measurements, due to the radiation exposure. Insertion torque values (ITV), measured intraoperatively, and resonance frequency analysis (RFA), performed postoperatively, have been used to evaluate primary stability (Lozano‐Carrascal et al., 2016), but these procedures cannot be performed preoperatively.

Speed of sound (SOS) values depend on the properties of the medium through which ultrasound propagates and correlates with the stiffness coefficient and mass density (Grodin et al., 2012; Sasso, Haiat, Yamato, Naili, & Matsukawa, 2008). Therefore, SOS is used clinically to evaluate bone quality in the field of orthopedics (Foldes, Rimon, Keinan, & Popovtzer, 1995; Njeh, Kuo, Langton, Atrah, & Boivin, 1997). The transverse ultrasound transmission device uses two types of transducers: the first is used as an emitter, while the second is used as a receiver. SOS values measured with this type of device have been shown to correlate with BMD values of the cancellous bone in the mandible (Al Haffar et al., 2006) and with the degree of calcification (Al‐Nawas et al., 2008). However, this type of device requires sufficient space in the mouth, and it is difficult to apply to the maxillary bone.

On the other hand, an axial ultrasound transmission device, which uses only one transducer, is a novel method for evaluation of bone quality for the SOS of cortical bone (cSOS). Several investigators have studied using cSOS in vivo (Grodin et al., 2012; Haiat, Naili, Ba Vu, Desceliers, & Soize, 2011). cSOS values measured by this type of device correlated with BMD values and porosity (Po) values of cortical bone in the radius. Thus, this type of device can evaluate bone quality (Bossy et al., 2004; Foldes et al., 1995). BMD values of cortical bone in the area for implantation correlated with the implant stability quotient (ISQ) values measured by RFA (Munakata, Shiota, Tetsumura, Tachikawa, & Kasugai, 2005). Furthermore, ITV measured when the miniscrews were inserted correlated with the BMD values of cortical bone (Marquezan et al., 2012). Therefore, it has been suggested that cSOS values measured using an axial transmission device in the area targeted for implantation hold potential for the preoperative prediction of primary stability.

The aim of this study was to investigate the clinical utility of an axial transmission device in preoperative measurement of bone quality for dental implantation, by clarifying the relationship between cSOS values, ITV, and ISQ values in porcine femur bone.

## 2 MATERIALS AND METHODS

This study used 11 porcine femurs from which the soft tissue had been removed. All bones were maintained at −20 °C until required, and all experiments were performed after the bones had been defrosted at room temperature.

### 2.1 Ultrasound measurement

**Figure 1:**
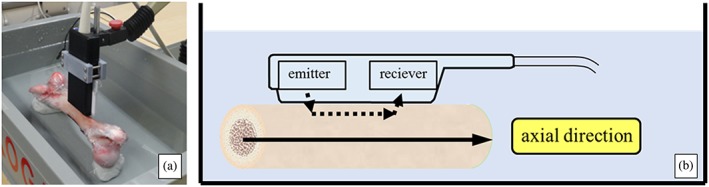
a, b. Axial transmission device and porcine bone sample used for this study. Cortical bone speed of sound measurements were performed in room temperature water

**Figure 2:**
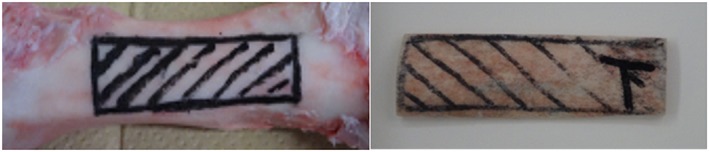
The region of cortical bone speed of sound measurements and cortical bone sample

Ultrasound measurements were performed using the axial transmission device (Furuno Electric, Hyogo, Japan). This device can calculate cSOS value from the propagation time difference and the propagation distance by irradiating a pulse with the center frequency 3.0 MHz from the emitter and receiving a leaky surface wave propagated through cortical bone with receiver. cSOS values, propagated from the proximal to the distal region of the bone, were measured at room temperature in a container filled with water (Figure 1a,b). Ultrasound measurements were repeated many times without changing the position and angle, and the average of 10 times drawing accurate waveform was calculated as the average value of cSOS. Eleven cortical bone samples were obtained from the region where ultrasound measurements were performed, using a precision diamond band saw (BS‐300CP; Exakt, Oklahoma City, OK, USA; Figure 2).

### 2.2 X‐ray microcomputed tomography

The 3‐D microarchitecture of each cortical bone sample was obtained by high resolution X‐ray microcomputed tomography (SKYSCAN 1176; Skyscan, Kontich, Belgium). X‐ray microcomputed tomography was performed with a pixel size of 18.0 mm and a Cu + Al filter (90 kV, 303 μA). The postprocessing software (CT‐An; Skyscan) was used to obtain the cortical BMD and Po of the bone sample region (5.0 × 20 × 1.5 mm) where ultrasound was propagated.

### 2.3 ITV measurement

**Figure 3:**
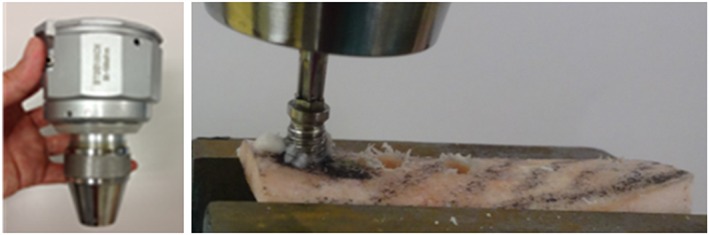
Digital torque gauge and regions of implantation. Ultrasound propagated the distal parts of the bone samples, and implants were inserted into the proximal, middle, and distal part of these parts

Thirty‐three titanium implants (Brånemark® Ti Unite Mark III, diameter 3.75 mm, length 8.00 mm; Nobel Biocare, Kloten, Switzerland) were inserted into the proximal, middle, and distal part of the bone samples; that is, three implants were placed per bone sample, using a series of drills. The ITV were obtained with a digital torque gauge (TOHNICHI, Tokyo, Japan; Figure 3). The mean ITV were calculated for each bone sample. Cortical bone thickness (CbTh) of the area for implantation was measured for all 33 implants using a micrometer (MDC‐255X; Mitutoyo, Kanagawa, Japan).

### 2.4 ISQ measurement

**Figure 4:**
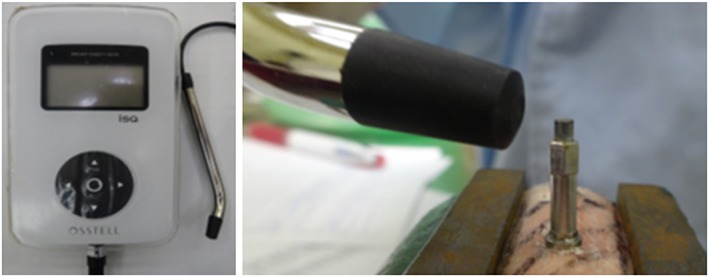
The wireless Osstell device and implant stability quotient measurements. These measurements were performed twice each in two perpendicular directions (mesio‐distal and bucco‐lingual)

A type 1 Smartpeg™ (Osstell, Gothenburg, Sweden) was attached to each implant. The RFA measurements were performed using a wireless Osstell device (Osstell® Mentors; Integration diagnostics AB), and ISQ values were obtained (Figure 4). These measurements were performed twice each in two perpendicular directions (mesio‐distal and inside‐outside), and the mean ISQ values were calculated.

### 2.5 Statistical analysis

All values were statistically analyzed by Spearman rank‐order correlation and level of significance (*p*), with the *p* level set at 5%.

## 3 RESULTS

### 3.1 Statistical correlation between parameters

#### 3.1.1 Between cSOS and BMD, Po

The mean cSOS value was 3962 m/s (min: 3642 m/s; max: 4190 m/s; standard deviation: 173.45).

**Figure 5:**
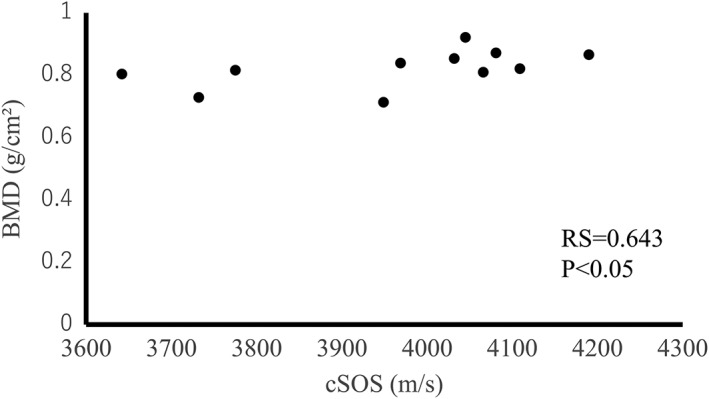
Relationship between the cortical bone speed of sound (cSOS) and bone mineral density (BMD) of each bone

**Figure 6:**
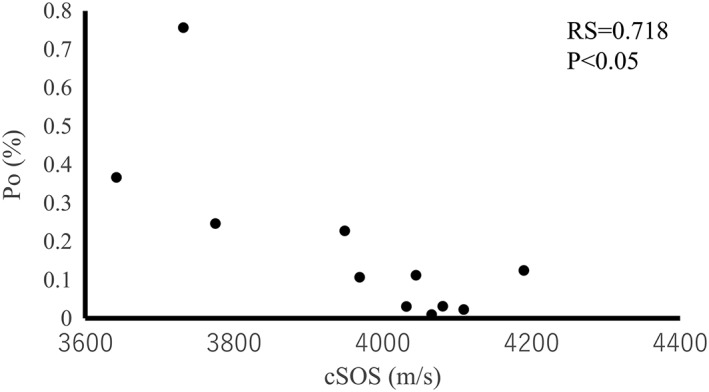
Relationship between the cortical bone speed of sound (cSOS) and porosity (Po) of each bone

The mean BMD and Po values were 0.822 g/cm2 (min: 0.713 g/cm2; max: 0.922 g/cm2; standard deviation: 0.061) and 0.185% (min: 0.0099%; max: 0.36619%; standard deviation: 0.22), respectively. The cSOS of each bone correlated with the BMD (RS = 0.645, *p* < .05) and Po (RS = −0.718, *p* < .05; Figures 5 and 6). These results revealed that cSOS reflects the bone quality of cortical bone.

#### 3.1.2 Between cSOS and ITV, CbTh

The mean ITV were 37.95 Ncm (min: 19.17 Ncm; max: 59.97 Ncm; standard deviation: 12.41).

**Figure 7:**
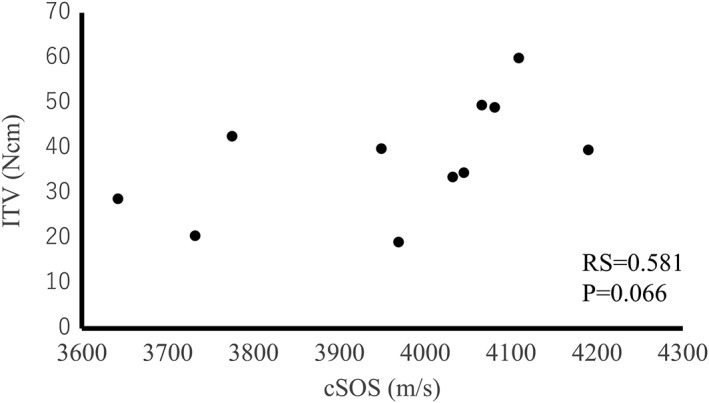
Relationship between the cortical bone speed of sound (cSOS) of each bone and the insertion torque values (ITV)

**Figure 8:**
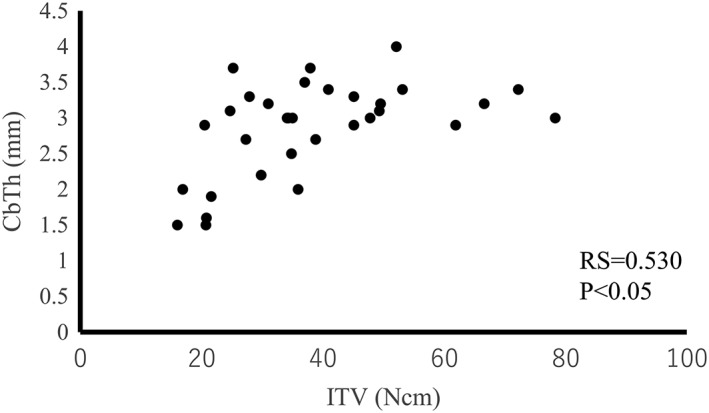
Relationship between insertion torque values (ITV) of each implant and the cortical bone thickness (CbTh) of the area for implantation

**Figure 9:**
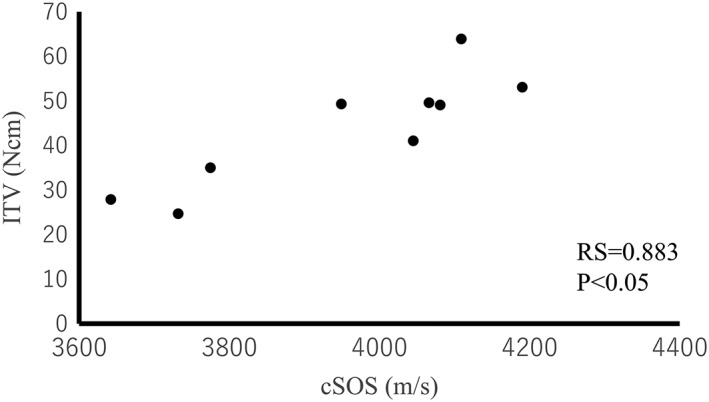
Relationship between the cortical bone speed of sound (cSOS) of each bone and the insertion torque values (ITV) in cases where cortical bone thickness ranged from 3.0 to 3.5 mm

The cSOS of each bone did not correlate with ITV for all of the bone samples (RS = 0.581, *p* = .066; Figure 7). On the other hand, the ITV of each implant correlated with the CbTh of the area for implantation (RS = 0.530, *p* < .05; Figure 8). Thus, ITV were strongly affected by CbTh. However, when the CbTh ranges from 3.0 to 3.5 mm, ITV are correlated with cSOS (RS = 0.883, *p* < .05; Figure 9). These results suggest that ITV are different even for the same thickness of cortical bone, and that difference may reflect cortical bone quality. Therefore, cSOS reflecting cortical bone quality may be clinically useful as a preoperative diagnosis of implants.

#### 3.1.3 Between cSOS and ISQ

**Figure 10:**
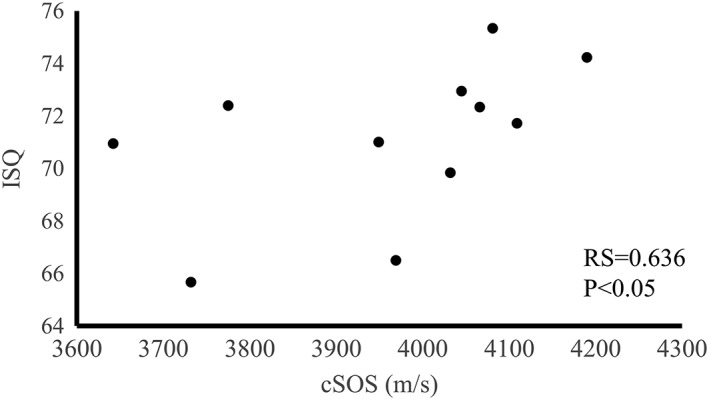
Relationship between the cortical bone speed of sound (cSOS) and the mean implant stability quotient (ISQ) of each bone

The mean ISQ values were 71.17 (min: 65.67; max: 75.33; standard deviation: 2.95). The cSOS of each bone correlates with ISQ (RS = 0.636, *p* < .05; Figure 10) for all of the bone samples. ISQ has become common to clinically evaluate implant stability (Meredith, Alleyne, & Cawley, 1996), and it facilitates the decision about whether immediate loading is feasible (Fischer, Bäckström, & Sennerby, 2009; Glauser et al., 2004). However, ISQ measurement cannot be used for preoperative diagnosis. This result suggests that there is a possibility that it can be estimated preoperatively whether or not immediate loading can be made by measuring cSOS.

## 4 DISCUSSION

To the best of our knowledge, this study is the first to report the relationship of cSOS values, determined using an axial ultrasound transmission device and dental clinical measurements. In this study, the correlation between cSOS and BMD, as well as between cSOS and Po, indicates that the axial transmission device used in this study, as well as in previous studies, reflects bone quality (Bossy et al., 2004). qCT has long been used to evaluate bone quality in the dental field (Kumar et al., 2012) but is unsuitable for repeated measurements due to the radiation exposure. On the other hand, ultrasound measurement requires no radiation exposure and is noninvasive (Mathieu, Chappard, Vayron, Michel, & Haiat, 2013). Thus, it is considered that ultrasound measurement can be used to evaluate the bone quality sequentially and to determine the appropriate time for implantation.

Previous studies have reported that ISQ correlates with the elastic modulus of the implant‐surrounding bone (Arai, Iwata, Saratani, Tanaka, & Kawazoe, 2007), and thus cSOS may correlate with ISQ, as it reflects bone mechanical properties. This result cSOS correlated with ISQ indicates that cSOS determined using an axial transmission device can predict ISQ preoperatively for determination of whether immediate loading can be performed. Moreover, cSOS did not correlate with ITV, except when CbTh was in the range of 3.0 to 3.5 mm. These results suggest that ITV are different even for the same thickness of cortical bone and that difference may reflect cortical bone quality, and cSOS reflects cortical bone quality exactly.

The axial transmission device used in this study allows medical clinicians to evaluate bone quality of the long bone, femur, and tibia, and so on (Daugschies, Rohde, Glüer, & Barkmann, 2014; Xu, Ta, He, Qin, & Wang, 2014). Therefore, miniaturization of the device and further studies with human jawbone are needed for this device to be used in the oral cavity. Furthermore, the soft tissue was removed from the bone samples used in this study. Therefore, these samples do not reflect the bone in a clinical situation, and the effect of the oral mucosa remains unclear.

However, evaluating bone quality of the area targeted for implantation using an axial ultrasound transmission device used in this study can predict the primary stability, preoperatively. These findings show that cSOS is valuable for evaluating bone quality.

## 5 CONCLUSION

cSOS reflects the cortical bone quality, suggesting the possibility of being clinically useful as a preoperative diagnosis of the implant in an ex vivo model.
